# Epidemiology of hand, foot and mouth disease in China, 2008 to 2015 prior to the introduction of EV-A71 vaccine

**DOI:** 10.2807/1560-7917.ES.2017.22.50.16-00824

**Published:** 2017-12-14

**Authors:** Bingyi Yang, Fengfeng Liu, Qiaohong Liao, Peng Wu, Zhaorui Chang, Jiao Huang, Lu Long, Li Luo, Yu Li, Gabriel M. Leung, Benjamin J. Cowling, Hongjie Yu

**Affiliations:** 1 WHO Collaborating Centre for Infectious Disease Epidemiology and Control, School of Public Health, Li Ka Shing Faculty of Medicine, The University of Hong Kong, Hong Kong Special Administrative Region, China; 2 These authors contributed equally to this work; 3 Division of Infectious Disease, Key Laboratory of Surveillance and Early-warning on Infectious Disease, Chinese Centre for Disease Control and Prevention, Beijing, China; 4 Department of Epidemiology and Statistics, Public Health School, Tongji Medical College, Huazhong University of Science and Technology, Wuhan, China; 5 Department of Epidemiology and Biostatistics, West China School of Public Health, Sichuan University, Chengdu, China; 6 These authors are joint senior authors; 7 School of Public Health, Fudan University, Key Laboratory of Public Health Safety, Ministry of Education, Shanghai, China

**Keywords:** HFMD, epidemiology, periodicity, case-fatality

## Abstract

Hand, foot and mouth disease (HFMD) is usually caused by several serotypes from human enterovirus A species, including enterovirus 71 (EV-A71) and coxsackievirus A16 (CV-A16). Two inactivated monovalent EV-A71 vaccines have been recently licensed in China and monovalent CV-A16 vaccine and bivalent EV-A71 and CV-A16 vaccine are under development. **Methods**: Using notifications from the national surveillance system, we describe the epidemiology and dynamics of HFMD in the country, before the introduction of EV-A71 vaccination, from 2008 through 2015. **Results:** Laboratory-identified serotype categories, i.e. CV-A16, EV-A71 and other enteroviruses, circulated annually. EV-A71 remained the most virulent serotype and was the major serotype for fatal cases (range: 88.5–95.4%) and severe cases (range: 50.7–82.3%) across years. Except for 2013 and 2015, when other enteroviruses were more frequently found in mild HFMD (48.8% and 52.5%), EV-A71 was more frequently detected from mild cases in the rest of the years covered by the study (range: 39.4–52.6%). The incidence rates and severity risks of HFMD associated with all serotype categories were the highest for children aged 1 year and younger, and decreased with increasing age. **Discussion/conclusion**: This study provides baseline epidemiology for evaluation of vaccine impact and potential serotype replacement.

## Introduction

Hand, foot and mouth disease (HFMD) causes a substantial disease burden in the Asia-Pacific region particularly in young children below 5 years of age [1,2]. HFMD is usually caused by enteroviruses (EV), which comprise in total 12 species [3]. Two serotypes from the A species, namely EV-A71 and coxsackievirus A16 (CV-A16), are generally considered as the most common causative pathogens for HFMD [1]. EV-A71 is the most frequently identified serotype among both severe and fatal cases, and has caused several outbreaks involving severe neurological symptoms in Spain and France in 2016 [1,2,4,5]. Aside from these two serotypes, other serotypes of the A species (i.e. CV-A2–8, 10, 12) and occasionally B species (i.e. CV-B4, echovirus 7, 9 and 11) have also been reported to cause HFMD and subsequent severe complications [6,7]. Particularly CV-A6 has been emerging and caused outbreaks worldwide in 2013 [4,5,7,8].

Currently there is no specific treatment for HFMD. Two inactivated monovalent EV-A71 vaccines were recently licensed in China [9], which showed high efficacy (94.8–97.4%) against EV-A71-associated HFMD but no cross-protection against HFMD caused by CV-A16 or other serotypes in children (6 to 35 months [10] and 6 to 71 months of age [11]). Monovalent CV-A16 vaccine and bivalent EV-A71 and CV-A16 vaccine are under development. Monitoring and modelling the vaccine impact and potential serotype replacement will therefore be critical in the post EV-A71/CV-A16 vaccine era. Currently, evidences on serotype-specific and individual age-patterns’ disease burden, which may inform possible transmission routes, are not well documented. We aimed to provide a baseline characterisation, before the introduction of EV-A71 vaccination, of the epidemiology and dynamics of HFMD by evaluating incidence rates, severity profile and periodicity using notifications from the national surveillance system in China.

## Methods

### Data sources

Data on HFMD cases from 1 January 2008 to 31 December 2015 were obtained from the National Surveillance of Notifiable Infectious Disease Programme (NSNIDP), which covered minimum 98% of hospitals at county level and 94% of health institutions at township/community level [12]. HFMD became a notifiable infectious disease in China on 2 May 2008 before which HFMD cases were voluntarily reported to the Chinese Center for Disease Control and Prevention (China CDC) [1]. From 2008 onwards, patients clinically diagnosed with HFMD were required to be reported to the NSNIDP, and basic demographic and diagnostic information was collected through online reporting. HFMD was diagnosed if a patient had skin papular or vesicular rash on hands, feet, mouth or buttocks, with or without fever [1,13]. Cases were categorised as severe if suffering any cardiopulmonary or neurological complications, otherwise as mild. The diagnostic criteria and details in case classifications have been described in previous studies [1,13].

Virological surveillance was conducted for a sub-sample of the cases from the NSNIDP. From June 2009 onwards, specimens (including throat swabs, rectal swabs and faecal samples, vesicular fluid, or cerebrospinal fluid) were collected from the first five mild cases and all severe cases of HFMD every month from each county/district. Prior to this period, the specimens were collected on a weekly basis at provincial level. Specimens were tested by virus isolation, reverse transcription PCR (RT-PCR) or real-time PCR and only positive results were reported to the NSNIDP with virus categorisation as EV-A71, CV-A16, and other EVs [1]. The information on the total number of cases tested is therefore unavailable. We defined cases with positive laboratory results as laboratory-confirmed cases, otherwise as probable cases.

### Data analysis

#### Periodicity of serotype-specific hand, foot and mouth disease cases

To analyse the periodicity of serotype-specific HFMD cases, we conducted wavelet analysis with weekly time series of laboratory-confirmed HFMD cases infected with EV-A71, CV-A16 and other EVs using the Morlet function respectively [14,15]. We first performed logarithmic transformation of the weekly number of HFMD cases associated with EV-A71, CV-A16 and other EVs respectively, and then normalised the data to have zero mean and unit variance. To reduce edge effects, we padded the time series with excess zeros. We used phase angles at 1 year period and the phase difference between EV-A71 and CV-A16 or other EVs was transformed by mod (difference + 540, 360)  – 180, so that all phase differences would be constrained within ±18° [14]. In the wavelet analysis, we only used cases reported since July 2009 which corresponded to a short time after the current virological surveillance was launched on 4 June. This was to avoid the potential for systematic differences to be introduced by the different surveillance methods, and to also avoid the potential impact caused by the transition period in the first days of the current surveillance [1]. 

#### Analyses by serotype

We analysed the serotype distributions during the study period using the severity-stratified monthly proportions of laboratory-confirmed HFMD cases infected with EV-A71, CV-A16 and other EVs at national level.

#### Incidence rates and severity risks of hand, foot and mouth disease

**Figure 1:**
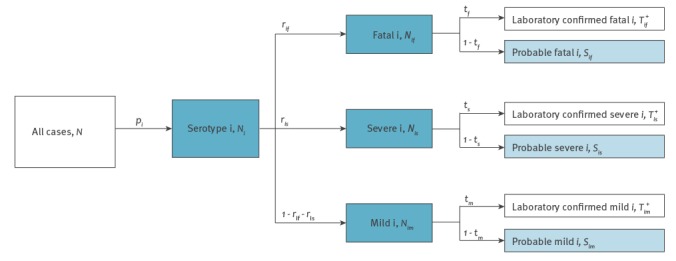
Schematic model framework to illustrate estimation of serotype-specific notifications of hand, foot and mouth disease and risks of severe events, mainland China, 2008–2015

We estimated the person-time incidence rate of HFMD by dividing the number of all notified (probable and laboratory-confirmed) HFMD cases by the total number person-time observed during the corresponding period. The case-severity risk (CSR), case-fatality risk (CFR) and severity-fatality rate (SFR) were defined as (i) the number of severe and fatal cases divided by the number of all cases, and (ii) the number of fatal cases divided by the number of all cases, and (iii) the number of fatal cases divided by the number of severe and fatal cases, respectively. We developed a Bayesian model (Figure 1), which accounts for the disproportional severity distribution in laboratory-confirmed cases, to estimate the incidence rates and severity risks of notified HFMD cases infected with EV-A71, CV-A16 or other EVs.

*N*: all reported hand, foot and mouth disease cases; *p_i_*: probability that a reported case**was caused by either enterovirus 71, or coxsackievirus A16, or other enteroviruses (* i* = 1, 2, and 3 respectively); *r_ij_*: probability of occurrence of cases infected with serotype *i* and severity *j* (for mild (*m*), severe (*s*) and fatal (*f*) respectively); *S_ij_*: probable cases with serotype *i* and severity* j*; *t_j_*: probability for a case with severity *j* of a specimen being sent for laboratory testing and having a positive result; *T^+^_ij_*: true number of laboratory-confirmed cases caused by serotype i with severity *j*.

Data were obtained from national notifications of cases with hand, foot and mouth disease in mainland China. Numbers in white boxes can be derived from the notification data while numbers in blue boxes will be estimated from the Bayesian model.

The CFRs, CSRs and SFRs for EV-A71, CV-A16 or other EVs were estimated simultaneously. We assumed a reported case was caused by either EV-A71, or CV-A16, or other EVs with a probability *p_i_* with *i* = 1, 2, and 3 respectively. The probability of occurrence of cases infected with serotype *i* and severity *j* (for mild (*m*), severe (*s*) and fatal (*f*) respectively) is *r_ij_*. For cases with severity *j*, we assumed that the probability of a specimen being sent for laboratory testing and having a positive result was *t_j_*. The true number of laboratory-confirmed cases caused by serotype *i* with severity* j* (*T^+^_ij_*) and the probable cases with severity *j* *(S_j_)* were modelled as sub-samples of all reported HFMD cases (*N*) following a Poisson distribution:

$T_{ij}^{+}\;\sim \;Poisson\;(Nt_{j}r_{ij}p_{i})$

$S_{j}\;\sim \;Poisson\;(N\sum_{i}\left(1-t_{j}\right)r_{ij}p_{i})$

We specified flat priors for all parameters. We estimated the parameters by year and by age group respectively.

All analyses were conducted using R (version 3.2.3; R Foundation for Statistical Computing) and MATLAB (version 8.4.0; MathWorks Inc.) software.

## Results

**Figure 2:**
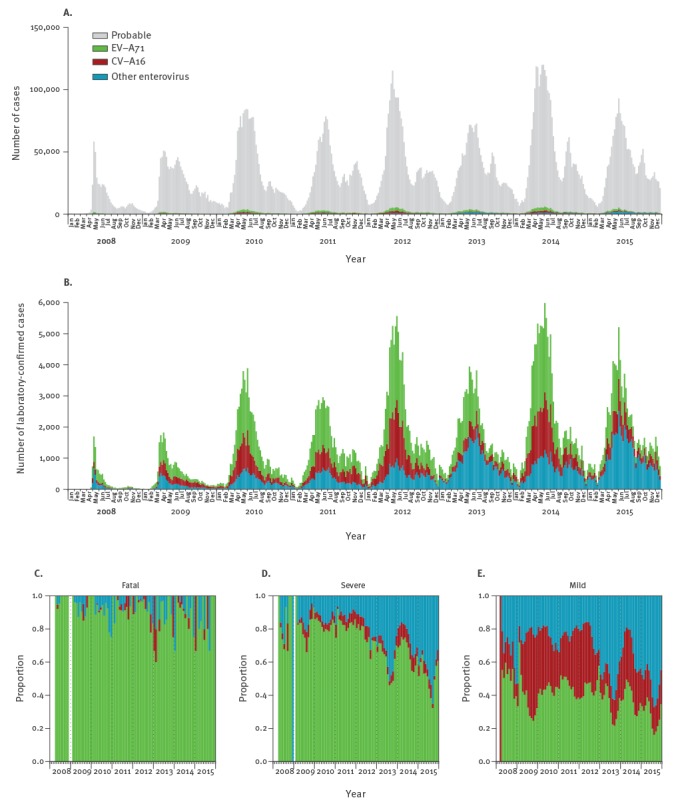
Notifications of probable and laboratory-confirmed hand, foot and mouth disease (HFMD) in mainland China, 2008–2015

In total, 13.7 million probable and laboratory-confirmed HFMD cases were reported from 2008 through 2015, among which 123,261 (0.90%) and 3,322 (0.02%) were severe and fatal cases, respectively (Table, Figure 2). Nationally (especially in the north), the annual number of cases peaked around the month of June with a subsequent decrease, followed by a milder re-increase during the autumn (September to October) before decreasing further (Figure 2). Among laboratory-confirmed cases, the proportions of severe and fatal cases were higher (9.92% (57,248/577,087; 95% confidence interval (CI): 9.84–10.00) and 0.40% (2,308/577,087; 95%CI: 0.38–0.42) respectively) than that among probable cases (0.50% (66,013/13,109,898; 95%CI: 0.50–0.51) and 0.008% (1,014/13,109,898; 95%CI: 0.007–0.008) respectively) (Table). The median age for all notified cases was 2.1 years (interquartile range (IQR): 1.4–3.5 years) and for fatal cases 1.6 years (IQR: 1.0–2.3 years) (Table). The male to female ratio was 1.6:1 for all notified HFMD cases. The age and sex distributions among probable and laboratory-confirmed cases were similar.

**Table:** Characteristics of probable and laboratory-confirmed hand, foot and mouth disease cases in mainland China, 2008–2015 (n = 13,686,985 cases)

Disease severity	Mild	Severe	Fatal	Total
**Number of cases**	13,560,402	123,261	3,322	13,686,985
**Median age, years (range)**	2.2 (1.4–3.5)	1.9 (1.1–2.8)	1.6 (1.0–2.3)	2.1 (1.4–3.5)
**Male, number (%)**	8,363,174 (61.7)	79,296 (64.3)	2,156 (64.9)	8,444,629 (61.7)
**Case category**				
Probable cases, number (%)	13,042,871 (96.2)	66,013 (53.6)	1,014 (30.5)	13,109,898 (95.8)
Laboratory-confirmed cases, number (%)	517,531 (3.8)	57,248 (46.4)	2,308 (69.5)	577,087 (4.2)
EV-A71, number (% among laboratory-confirmed cases)	207,076 (40.0)	42,236 (73.8)	2,136 (92.5)	251,448 (43.6)
CV-A16, number (% among laboratory-confirmed cases)	140,225 (27.1)	3,285 (5.7)	43 (1.9)	143,553 (24.9)
Other enterovirus, number (% among laboratory-confirmed cases)	170,230 (32.9)	11,727 (20.5)	129 (5.6)	182,086 (31.6)

CV-A16: coxsackievirus A16; EV-A71: enterovirus 71.

A: Weekly notifications of probable and laboratory-confirmed cases with HFMD; B: Weekly notifications of laboratory-confirmed cases with HFMD by serotype; C–E: Monthly proportions of notifications of laboratory-confirmed fatal, severe and mild cases with HFMD by serotype.

### Periodicity of hand, foot and mouth disease cases

**Figure 3:**
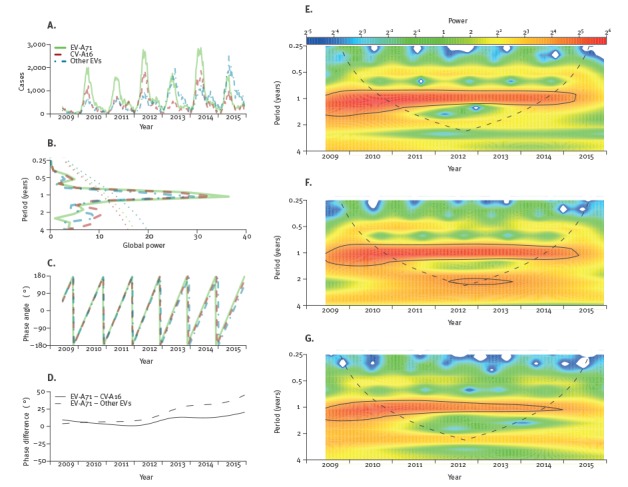
Wavelet analyses for time series of serotype-specific notifications of hand, foot and mouth disease in mainland China, 2009–2015

CV-A16: coxsackievirus A16; EV: enterovirus.

Results from the wavelet analysis suggested that both global, i.e. the average throughout the study period (Figure 3 B), and local, i.e. for each time step examined, (Figure 3 E–G), powers were the largest at the period of 1 year, suggesting HFMD cases attributed to the three categorisations of serotypes showed a significant annual periodicity.

A: Weekly numbers of laboratory-confirmed cases by serotype. B: Global wavelet power estimates by serotype. C: Phase angles at scale of 1 year by serotype. D: Phase difference between EV-71 and CV-A16 and other EVs based on phase angles. E–G: Local wavelet power spectrum for EV-A71 (E), CV-A16 (F) and other EVs (G) (solid circles indicate boundary of statistical significance; dashed lines are ‘core-of-influence’ estimates that may be affected by the edge effect and therefore should be interpreted with caution).

Results also suggested a weaker semi-annual and biennial periodicity for all three serotype categories (Figure 3). The sensitivity analysis using daily-unit time series showed significant semi-annual, annual and biennial periodicity for all three serotype categories while the power of the annual periodicity was still the strongest throughout the study period (data not shown). Phase differences between EV-A71 and CV-A16 or other EVs became more obvious after 2013 (Figure 3 C-D) and results from the wavelet coherence analysis also suggested a small lead of EV-A71 after 2013 (data not shown).

### Serotype distributions

Among 577,087 laboratory-confirmed HFMD cases, 43.6% and 24.9% were associated with EV-A71 and CV-A16 respectively (Table). EV-A71 was the predominant serotype among laboratory-confirmed fatal cases, accounting annually for 88.5% (77/87 in 2015) to 95.4% (418/438 in 2012) of such cases during the study period (Figure 2 C). Annually 50.7% (2,549/5,028 in 2015) to 82.3% (6,799/8,261 in 2011) of laboratory-confirmed severe HMFD cases were infected with EV-A71, while 11.9% (980/8,261 in 2011) to 43.9% (2,207/5,028 in 2015) were infected by other EVs (Figure 2 D). In 2013 and 2015, 48.8% (40,034/81,992) and 52.5% (48,859/93,141) of laboratory-confirmed mild HFMD cases were caused by other EVs, which is more than the percentages of such cases, which were due to EV-A71 and CV-A16 respectively (Figure 2 E).

### Incidence, severity and mortality rates of hand, foot and mouth disease

**Figure 4:**
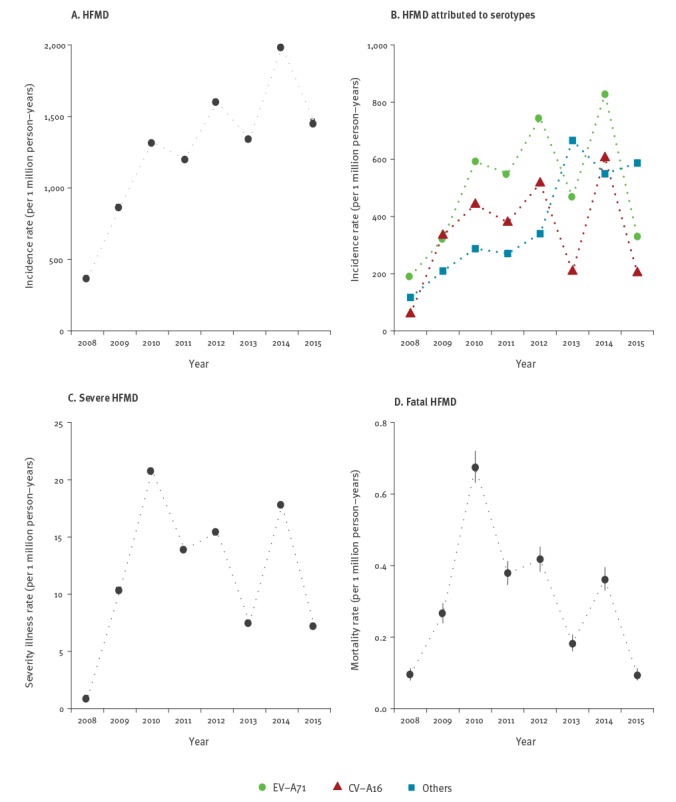
Annual incidence rates of notified hand, foot and mouth disease in mainland China, 2008–2015

CV-A16: coxsackievirus A16; EV-A71: enterovirus 71; HFMD: hand, foot and mouth disease.

The mean annual incidence rate of HFMD was around 1,270 per 1 million person-years during the study period, and the highest annual incidence rate was around 1,980 per 1 million person-years in 2014 (Figure 4 A). The mean annual severe illness rate and mortality rate were 11.8 (95% CI: 11.7–11.8) per 1 million person-years and 0.308 (95% CI: 0.307–0.310) per 1 million person-years, respectively, and both were at their highest in 2010 (Figure 4 C-D).

Vertical solid lines represent 95% confidence intervals or credibility intervals of the rates, and may not be visible due to a narrow interval between the lower and upper bound.

The incidence rates of HMFD cases attributed to EV-A71, CV-A16 and other EVs were estimated to be 522 (95% credible interval (CrI): 517–527), 351 (95% CrI: 347–356) and 398 (95% CrI: 393–402) per 1 million person-years, respectively, during the study period. The incidence rate of EV-A71 was the highest among all serotype categories during the study period except for the years of 2013 and 2015, when the incidence rate of HFMD associated with other EVs was the highest with 654 (95% CrI: 650–659) and 766 (95% CrI: 762–771) per 1 million person-years, respectively (Figure 4 B).

**Figure 5:**
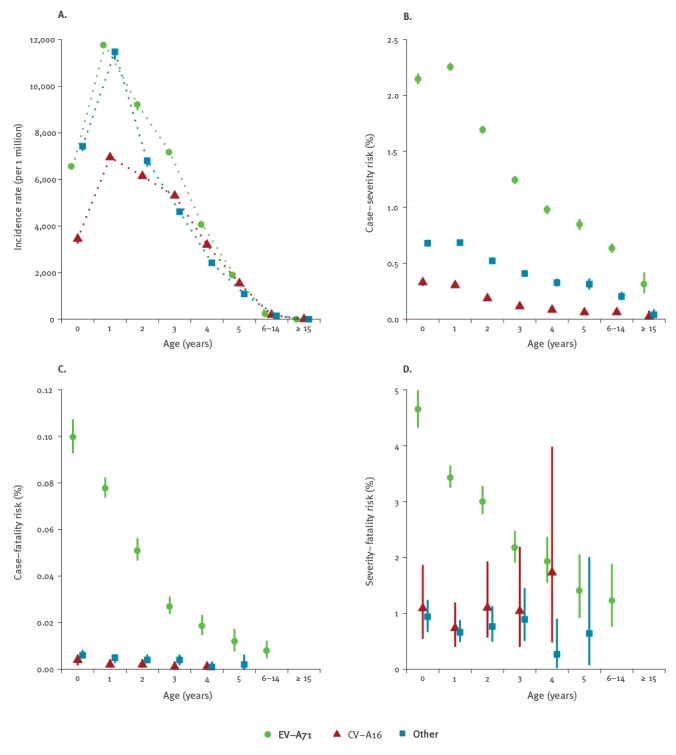
Age-specific incidence rate (Panel A), case-severity risk (Panel B), case-fatality risk (Panel C) and severity-fatality risk (Panel D) of notified hand, foot and mouth disease cases by serotype in mainland China, 2008–2015

Incidence rates were highest in children at 1 year of age, and declined with older age (Figure 5). The incidence rate of HFMD notifications was higher in the south compared with the north of China during the study period (1,190 and 640 per 1 million person-years, respectively), while the age pattern of incidence rates in the north and south were estimated to be similar to the overall pattern shown in Figure 5.

Vertical lines represent 95% credibility intervals of the estimate, and may not be visible due to a narrow interval between the lower and upper bound.

### Severity and fatality risks of hand, foot and mouth disease

The overall CSR and CFR were estimated to be 0.925% (95% CrI: 0.920–0.930%) and 0.024% (95% CrI: 0.023–0.025%) respectively. The CSR and CFR for EV-A71 were estimated to be 1.74% (95% CrI: 1.72–1.75) and 0.055% (CrI: 0.053–0.057). The CSR and the CFR were substantially higher among cases associated with EV-A71 than with CV-A16 or other EVs (Figure 5 B-D). CSR peaked at 1 year of age with estimation of 2.26% (95% CrI: 2.23–2.29), 0.30% (95% CrI: 0.29–0.32) and 0.69% (95% CrI: 0.67–0.70) for each type of EV considered, while CFR peaked at under 1 year of age with 0.099% (95% CrI: 0.093–1.07), 0.004% (95% CrI: 0.002–0.006) and 0.006% (95% CrI: 0.005–0.008) per EV category respectively. After peaking, both the CSR and the CFR decreased along with age increase (Figure 5 B-C). The SFR of EV-A71 associated cases declined with age while no substantial differences in the SFR were observed across age groups for cases infected with CV-A16 and other EVs (Figure 5 D).

## Discussion

EV-A71, CV-A16 and other EVs were detected annually from 2008 through 2015 in mainland China, while a weaker half-year periodicity was also suggested by our results. Currently, little evidence is available about the underlying mechanism of autumn waves, which occur after the main annual epidemic peak around June. In addition, our finding on the periodicity of HFMD epidemics in mainland China is not particularly consistent with the general view of 2-year periodicity for all serotypes, which is reported elsewhere [2]. Phase differences between serotypes were not obvious especially before 2013, suggesting a similar periodicity and small difference in the timing of epidemics across serotypes, which is consistent with the limited cross-immunity between different serotypes [16]. No major reduction in notified HFMD incidence rates was observed in years following a larger epidemic, such as the years 2013 and 2015, indicating these large epidemics might not have depleted susceptible persons in the population probably due to a steady birth rate and alternating serotypes without cross-protection. Monovalent EV-A71 vaccines have recently been licensed in China, and are projected to substantially decrease the burden of HFMD caused by EV-A71 but not the burden caused by CV-A16 [16].

We found the incidence of HFMD associated with other EVs was the highest compared with EV-A71 and CV-A16 during 2013 and 2015. Due to limitations of our data we were not able to further evaluate individual contributions of other enterovirus serotypes, which in total accounted for 48.8% and 52.5% of mild cases in 2013 and 2015. Studies from seven provinces/prefectures reported that a median of 49% (IQR: 43–56%) of all EV test-positive specimens were due to CV-A6 in 2013 and 2015, indicating a wide emergence of CV-A6 in the country since 2013 [6,8,17-23]. The increasing contribution of other EVs in China since 2013 was also consistent with the worldwide (including Europe) outbreak of CV-A6 in 2013. EV-A71 remained the most virulent serotype in China accounting for 88.5–95.4% of laboratory-confirmed fatal cases [1,13], although it was responsible for fewer severe cases in 2015 even with a significantly higher CSR and CFR compared with CV-A16 and other EVs. We were not able to assess the distribution of EV-A71 subgenotypes in patients with different severity or in different age groups due to lack of data. Other studies reported that the most isolated EV-A71 strains belonged to the subgenotype C4, especially C4a clusters, during 2002 and 2013 in mainland China, while currently there is no conclusion on the association between EV-A71 subgenotype and disease severity [24-26]. Mass EV-A71 vaccination is not expected to substantially reduce the total number of HFMD cases because the vast majority of cases (99%) are mild, and more than half of the mild cases were due to CV-A16 and other EVs combined. However, most fatal and half of severe HFMD cases could be prevented by EV-A71 vaccination [11]. Given the potential threats from diverse EVs and varied patterns in co-circulation each year [27], a comprehensive virological surveillance on non-EV-A71 serotypes would therefore be critical in early warning for HFMD outbreaks, monitoring serotype replacement and informing potential multivalent vaccine development in the post EV-A71 vaccination period.

We found that incidence rates peaked at 1 year of age and declined with age for all serotypes, which is consistent with previous serological evidence [28]. Similar to other studies, children at 6 to 11 months of age had a much higher incidence (overall 31.9 per 1,000 person-years) compared with children younger than 5 months (overall 2.6 per 1,000 person-years), which is probably due to the waning maternal immunity [28]. Compared to e.g. school aged children, children aged 1 year or younger may have a lower intensity of contact with those of similar age due to their limited mobility, so the high incidence rate among this age group may suggest higher susceptibility due to lower immunity or/and possible transmission routes from contact with asymptomatic infectious adults or contaminated environment (i.e. water) [28]. Due to limited observations on fatal cases caused by CV-A16 and other EVs among children older than 5 years, we were not able to provide estimates of the CSRs and CFRs for the corresponding age groups (Figure 5). Our estimates of CFRs and CSRs showed a decreased trend with increasing age after 1 year of age for all serotypes, while estimates of CSRs and SFRs may be affected by varied practices in diagnosis of mild and severe cases across the country. EV-A71 and potential multivalent vaccines therefore should be recommended to children of younger ages, due to the high risk of infection and relatively higher severity risks. The peaking age of EV-A71 associated incidence rate is expected to shift to older age after mass vaccination due to vaccine-derived immunity waning and reduced population-level risk of EV-A71 infection, while age-pattern of infection of serotypes other than EV-A71 may remain unchanged [28]. CFR of EV-A71 were estimated as 0.055% (95% CI: 0.053–0.057) from our model, which is much lower than the estimate (1.8%; 95% CI: 1.2–2.7) from a systematic review using laboratory-confirmed EV-A71 cases as denominator [29]. Severity distributed disproportionally among laboratory-confirmed cases (Table), which may overestimate the CFR directly using laboratory-confirmed EV-A71 cases. In addition, our findings in the age and serotype profile of HFMD cases could aid to identify the population at high risk of the disease, especially for areas with recent HFMD outbreaks including Europe.

Our study has several limitations. First, we were not able to fully assess the roles of specific serotypes other than EV-A71 and CV-A16 in HFMD epidemics in China given the limited laboratory information on other EVs. Second, our results faced a common challenge for studies using data from surveillance on a self-limited disease with possible under-reporting or variations in diagnosis practices of cases, particularly for mild and subclinical cases. Moreover, the surveillance system was HFMD syndrome based, and enteroviruses which cause neurological symptoms but not HFMD may not be detected. In addition, we were not able to distinguish the cases with laboratory test-negative results from those without specimen collection, while the uncertainty of caused by the test-negative cases (e.g. false positives), especially for mild cases, might affect our estimates [30]. Finally, HFMD was voluntary reported before 2 May 2008 in mainland China, which may lead to underestimation of the incidence rates, severe illness rates and mortality rates estimated for 2008. Such voluntary report in early 2008 would however not affect our main findings, including the age profiles of HFMD cases as the contribution from cases reported during that period was limited (only 0.14% of all HFMD cases were reported before 2 May 2008).

## Conclusions

HFMD caused a substantial burden of disease in China during 2008 through 2015, while all serotypes circulated every year. The incidence rate and severity risk decreased with age for all serotypes categories. EV-A71 remained the most virulent serotype causing more severe and fatal cases, while contribution from other EVs increased among mild and severe cases in 2013 and 2015. Potential threats from EVs other than EV-A71 and CV-A16 were suggested, comprehensive virological surveillance on specific EV serotypes is therefore necessary to monitor the potential ecological impact of EV-71/CV-A16 vaccination.
